# Perinatal features of Prader-Willi syndrome: a Chinese cohort of 134 patients

**DOI:** 10.1186/s13023-020-1306-z

**Published:** 2020-01-21

**Authors:** Lili Yang, Qiong Zhou, Bo Ma, Shujiong Mao, Yanli Dai, Mingqiang Zhu, Chaochun Zou

**Affiliations:** 1 Department of Genetics and Metabolism, Children’s Hospital, Zhejiang University School of Medicine, National Clinical Research Center for Child Health, Hangzhou 310052, China; 2 Department of Endocrinology and Metabolism, Hangzhou Children’s Hospital, Hangzhou, China; 3 Department of Obstetrics and Gynecology, Tianjin Central Hospital of Gynecology Obstetrics, Tianjin, China; 4 Department of Pediatrics, Hangzhou First People’s Hospital, Zhejiang University School of Medicine , Hangzhou, China; 5 Department of Endocrinology, Children’s Hospital, Zhejiang University School of Medicine, National Clinical Research Center for Child Health, 3333 Binsheng Rd, Hangzhou 310052, China

**Keywords:** Prader-Willi syndrome, Feature, perinatal, Complication

## Abstract

**Background:**

Prader-Willi syndrome (PWS) is a rare and complex genetic disorder caused by lacking expression of imprinted genes on the paternally derived chromosome 15q11-q13 region. This study aimed to characterize the perinatal features of 134 Chinese individuals with PWS.

**Methods:**

This study included the patients of a PWS registry in China. Anonymous data of 134 patients were abstracted. Perinatal and neonatal presentations were analyzed, and compared between the two PWS genetic subtypes. We also compared the perinatal features of PWS patients with the general population and other previous reported large cohorts from France, UK and USA.

**Results:**

This study included 134 patients with PWS (115 patients with 15q11-q13 deletion and 19 with maternal uniparental disomy). Higher mean maternal age was found in this cohort (30.5 vs. 26.7), particularly in the maternal uniparental disomy (UPD) group (36.0 vs. 26.7) comparing with the general population. 88.6% of mothers reported a decrease of fetal movements. 42.5 and 18.7% of mothers had polyhydramnios and oligohydramnios during pregnancy, respectively. 82.8% of the patients were born by caesarean section. 32.1% of neonates had birth asphyxia, 98.5% had hypotonia and 97.8% had weak cry or even no cry at neonatal period. Feeding difficulty existed in 99.3% of the infants, 94.8% of whom had failure to thrive. 69.4% of the infants ever used feeding tube during hospitalization, however, 97.8% of them discontinued tube feeding after discharge. Maternal age and pre-pregnancy weight were significantly higher in the UPD group (both *P* < 0.05).

**Conclusions:**

Differential diagnosis of PWS should be highlighted if infants having following perinatal factors including polyhydramnios, decreased intrauterine fetal movements, caesarean section, low birth weight, feeding difficulty, hypotonia and failure to thrive. Higher maternal age may be a risk factor of PWS, especially for UPD. Further studies are needed for elucidating the mechanism of PWS.

## Author summary

Early diagnosis and tailored multidisciplinary treatment are extremely important for better life quality of the infants with Prader-willi syndrome (PWS). Genetic diagnosis for PWS is now easily available, in which diagnosis can be comfirmed in most patients within the first months of life even in prenatal period if obstetricians or neonatologists can recognize the perinatal features of PWS well. However, most patients still had a delayed diagnosis because the early sign of PWS was not recognized. Our study highlighted the perinatal features of a large cohort of Chinese patients with PWS, which will benefit for early diagnosis and treatment of PWS. We found the incidences of decreased fetal movement, polyhydramnios and delivery by caesarean section were high. Maternal age was also higher comparing with the general population. Neonatal features found in our cohort included low birth weight, birth asphyxia, failure to thrive, feeding difficulty, weak cry and hypotonia. We found mothers with UPD had higher maternal age and pre-pregnancy weight. Most patients required tube feeding during hospitalization, however tube feeding was discontinued by their parents after discharge at home. Nutrition deficiency was a serious problem in infants with PWS. Home tube-feeding education should be strengthened in parents of PWS patients in China.

## Introduction

Prader-Willi syndrome (PWS) is a rare and complex genetic disorder characterized by severe hypotonia and feeding difficulties in early infancy, followed by excessive eating and gradual development of morbid obesity in early childhood, together with a series of comorbidities including short stature, typical facial dysmorphism, psychomotor delay, behavioral abnormalities and cognitive impairment [1]. It is caused by an absence of imprinted genes expression on the paternally derived chromosome 15q11-q13 region. PWS was divided into several genetic subtypes: deletion of the paternal copy of 15q11–13 in about 65% of the cases, maternal uniparental disomy (UPD) of chromosome 15 in about 30%, imprinting center defect in less than 5% and very rare cases of translocation involving the chromosome 15q11-q13 region [2, 3]. Genetic diagnosis for PWS is now easily available, in which diagnosis can be made in most patients within the first months of life even in prenatal period if obstetricians or neonatologists can recognize the perinatal features of PWS well. Increased awareness among obstetricians and healthcare providers would promote earlier diagnosis and treatment of PWS by pediatricians/neonatologists [4]. Early diagnosis and tailored multidisciplinary treatment are extremely important for better life quality of the infants with PWS as they ensure comprehensive advice to prevent obesity and to stimulate cognitive and adaptive skills [4, 5].

It is imperative to make more obstetricians, pediatricians/neonatologists be aware of the perinatal features of PWS. Our study aimed to characterize the perinatal features in a cohort of Chinese individuals with PWS. We also compared the perinatal features of PWS patients with the general population and other previously reported large cohorts from France, UK and USA. This is the first large study on patients with PWS in China.

## Methods

### Subjects

This study is part of a project started by PWS Research Group from Children’s Hospital, Zhejiang University School of Medicine. The PWS Research Group established a PWS Registry for patients in China. The study was approved by Institutional Review Board of Zhejiang University School of Medicine (no. 2018-IRB-055). Written informed consents were obtained from all parents or the patients (above 8 years) registered in the PWS Registry.

We included 134 patients from the PWS Registry into this study. All of the patients were confirmed by molecular genetic diagnosis. MS-PCR or MS-MLPA was used for initial diagnosis. For those with PWS methylation pattern but non-deletion type after initial diagnosis, microsatellite linkage analysis was used to differentiate UPD or an imprinting defect. Perinatal and neonatal presentations were analyzed and compared between the two PWS genetic subtypes as well as general population [6–10] and data reported from other countries [5, 11–14]. The analysis excluded one female and six males diagnosed only by methylation test.

### Statistical analysis

All of the data were analyzed by the SPSS (version 16.0) and R3.3.1 software. The categorical variables were summarized using frequencies and continuous variables were expressed as mean ± SD scores. The statistical differences between groups were compared by student t-test for continuous variables and Pearson χ2 test for categorical variables. Fisher’s exact test was performed to analyze differences of categorical variables when the expected cell count was less than 5. We applied one way ANCOVA to compare maternal pre-pregnancy weight and BMI between UPD and Deletion groups adjusted by maternal age. *P* value or FDR *P* value values were all two-sided in this study with statistically significantly level of 0.05.

## Results

This study included 134 patients with PWS including 115 (85.8%) with 15q11-q13 deletion and 19 with maternal UPD (14.2%). The studied patients’ birth dated from October 1997 to August 2018; among them, 117 patients’ birth dated between January, 2009 and August 2018. Among these patients, 73 (54.5%) were boys (62 with deletions and 11 with maternal UPD) and 61 (45.5%) girls (53 with deletions and 8 with maternal UPD). The mean age of diagnosis was 31.75 ± 4.72 months with a range from 10 days to 17 years.

Higher maternal age was found in this cohort (30.5 vs. 26.7), particularly in the UPD group (36.0 vs. 26.7), when comparing with general population. 87.9% (109/124) of the mothers reported a decrease of fetal movements during pregnancy. 42.5 and 18.7% of mothers had polyhydramnios and oligohydramnios during pregnancy, respectively. 82.8% of the patients were born by caesarean section, significantly higher than 34.9% in 2014 [7]; only 17.2% of the patients were born by vaginal delivery (23/134), and 30% of them by forceps. 2.2% of mothers had hypertension during pregnancy, 5.2% had gestational diabetes and 9.7% had premature rupture of membranes (Table 1).

**Table 1:** Perinatal factors of Prader-Willi syndrome and comparison with the general population in China

Perinatal factors	Total (*N* = 134)	Deletion (*n* = 115)	UPD (*n* = 19)	Population statistics	*P* value*
Maternal variables					
Maternal age (year), mean ± SD (range)	30.5 ± 5.5 (18–46)	29.6 ± 5.0 (18.0–46.0)	36.0 ± 6.1 (21.4–44.5)	26.7 ± 4.3^[6]^	< 0.001
Maternal pre-pregnancy weight (kg), mean ± SD	55.8 ± 8.4	55.2 ± 8.3	59.9 ± 7.8	NA	0.047^**^
Maternal pre-pregnancy BMI (kg/m^2^), mean ± SD	21.7 ± 3.1	21.4 ± 3.0	23.3 ± 2.9	20.7 ± 3.1^[6]^	0.061^**^
Fetal movements, *n* (%)^a^: Decreased	109 (87.9%)	94 (87.0%)	15 (93.7%)	NA	0.69
Normal	14 (11.3%)	13 (11.3%)	1 (5.3%)	NA	–
Increased	1 (0.8%)	1 (0.9%)	0 (0.0%)	NA	–
Pregnancy hypertension, *n* (%)	3 (2.2%)	3 (2.6%)	0 (0.0%)	3.6%^[6]^	0.51
Gestational diabetes, *n* (%)	7 (5.2%)	7 (5.9%)	0 (0.0%)	8.1%^[6]^	0.31
Preeclampsia, *n* (%)	0 (0%)	0 (0.0%)	0 (0.0%)	0.52%^[6]^	–
Polyhydramnios, *n* (%)	57 (42.5%)	49 (42.6%)	8 (42.1%)	1–3%^[6]^	0.79
Oligohydramnios, *n* (%)	25 (18.7%)	23 (20.0%)	2 (10.5%)	0.4–4.0%^[6]^	0.49
Premature rupture of membranes, *n* (%)	13 (9.7%)	12 (10.4%)	1 (5.3%)	2–4%^[6]^	0.69
Mode of delivery, *n* (%): Vaginal	23 (17.2%)	19 (16.5%)	4 (21.1%)	65.1%^[7]^	–
Caesarean section	111 (82.8%)	96 (83.5%)	15 (78.9%)	34.9%^[7]^	0.24
Neonatal variables					
Preterm birth (< 37 weeks), *n* (%)	22 (16.4%)	19 (16.5%)	3 (15.8%)	7.1%^[8]^	0.91
Low birth weight (< 2.5 kg), *n* (%)	46 (34.3%)	40 (34.8%)	6 (31.6%)	7.2%^[9]^	0.79
Birth length (cm), mean ± SD: Boys	48.5 ± 3.2	48.6 ± 2.8	48.0 ± 4.7	46.9–54.0 ^[10]^	0.40
Girls	49.4 ± 3.7	49.2 ± 3.9	50.3 ± 1.3	46.4–53.2 ^[10]^	0.90
Birth asphyxia, *n* (%)	43 (32.1%)	39 (34.0%)	4 (21.0%)	6.3%^[6]^	0.30
Hospitalization at neonatal period: Yes, *n* (%)	127 (94.8%)	111 (96.5%)	107 (84.2%)	NA	0.06
Duration (day), mean ± SD (range)	17.0 ± 13.6 (0–90)	18.1 ± 14.0 (0–90)	12.3 ± 8.8 (0–28)	NA	0.87
Breast feeding, *n* (%)	15 (11.2%)	12 (10.4%)	3 (15.8%)	58.5%^[9]^	0.52
Feeding difficulty, *n* (%)	133 (99.3%)	114 (99.1%)	19 (100%)	NA	0.81
Feeding tube used, *n* (%)	93 (69.4%)	83 (72.2%)	10 (52.6%)	NA	0.11
Weak cry, *n* (%)	131 (97.8%)	113 (98.3%)	18 (94.7%)	NA	0.37
Hypotonia, *n* (%)	132 (98.5%)	114 (99.1%)	18 (94.7%)	NA	0.26
Failure to thrive, *n* (%)	127 (94.8%)	109 (94.8%)	18 (94.7%)	8.1%^[9]^	0.99

*UPD* Uniparental disomy, *BMI* Body mass index, *SD* Standard deviation, *NA* not available

a: Lacking the information of fetal movement in 10 patients; *: compared using student’s *t*-test for continuous variables and Pearson χ^2^ test between the deletion and UPD groups; **: compared with one way ANCOVA after adjusting maternal age

This cohort of patients had a higher rate of prematurity (16.4%) and low birth weight (34.3%) compared with 7.2 and 7.1%, respectively in China. The patients revealed a higher rate of birth asphyxia (32.1%) than the general population of neonates in 2014 reported by Ministry of Health of China (6.3%) [6]. 94.8% of the patients were hospitalized after birth with a median duration of 17 days (range: 0–90 days). 94.8% of the patients had failure to thrive which was greatly higher than the general population (8.1%) [9]. Only 11.2% of the patients were purely breastfed at the first three months of life, which is lower than the general population (58.5%) [9]. Feeding difficulty existed in 99.3% of the patients. 69.4% of the patients used feeding tube, however, 97.8% of them discontinued feeding tube and used silicone bottle, spoon and even a syringe for feeding at home. Only two of them continued using feeding tube after hospitalization. 98.5% of the patients had hypotonia and 97.8% had weak cry during neonatal period.

All maternal and neonatal variables were compared between the patients with deletion and UPD (Table 1). Higher maternal age and maternal pre-pregnancy weightwere noted in the UPD group than that in the deletion subgroup (both *P* < 0.05). However, the rates of hospitalization during neonatal period and feeding tube use were higher in the deletion group than that in the UPD group with marginal differences (*P* = 0.06 and *P* = 0.11, respectively). We also compared our study with other large cohorts including two from France, one from UK and two from the United States (Table 2) [5, 11–14]. Great similarities were found among these cohorts. Our cohort had a much higher rate of polyhydramnios than that of the French cohorts (both FDR *P* < 0.05) and USA cohort (FDR *P* < 0.001).

**Table 2:** Comparison with other reported large studies on perinatal variables in Prader-Willi syndrome

Perinatal variables	China (N = 134)	France (*N* = 86) (2006)^[11]^	United Kingdom (*N* = 46) (2008)^[12]^	France (*N* = 61) (2017)^[5]^	United States (*N* = 64) (2018)^[13]^	United States (*N* = 355) (2018)^[14]^
Mean maternal age (year): Deletion	29.6	29.3	31.4	31	28.7	29.2
UPD	36.0	36.4	37.9	38	36.7	35.2
Decreased fetal movements	109/124 (87.9%)	47.6%	67.4%	27%	85.6%	78%
Polyhydramnios	57 (42.6%)	26.7%*	28.3%	23.0%*	NA	18%**
Vaginal delivery	23 (17.2%)	41.8%	17.4%	32.7%	56.5%	45.4%
Caesarean section	111 (82.8%)	53.4%	52.2%	67%	42.1%	54.6%
Preterm < 37 weeks	22 (16.4%)	15%	37%	20%	31.7%	26%
Hypotonia	132 (98.5%)	96.5%	100%	NA	84.3%	99.7%
Feeding difficulty	133 (99.3%)	82.5%	100%	84.4%	96.8%	99%

*UPD* Uniparental disomy, *NA* not available, *: FDR *P* < 0.05, compared with the Chinese cohort; **: FDR *P* < 0.001, compared with the Chinese cohort

## Discussion

PWS can now be diagnosed at a very early age or even at prenatal period benefiting from the improved molecular diagnosis techniques and increased awareness of PWS features [5, 14–16]. Dobrescu et al. and Gold et al. reported that most patients still had a delayed diagnosis because of the early signs of PWS not recognized, inappropriate molecular diagnostic methods used and lacking of expertise in some hospitals and institutions [4, 17]. Early diagnosis permits early care and treatment, which may reduce the hospital stay and tube feeding duration, hence preventing growth retardation and early onset of obesity [5, 18–20]. To our knowledge, this is the first large cohort study on perinatal features of PWS patients in China.

Recognizing perinatal features of PWS will be helpful for early diagnosis and multidisciplinary care of affected infants. The results of our cohort showed high rates of polyhydramnios (42.5%), decreased fetal movements (87.9%), caesarean section (82.8%), low birth weight (32.8%), feeding difficulty (99.3%), hypotonia (98.5%) and failure to thrive (94.5%). The results were similar to previously reported cohorts from the UK, France and USA. Dudley et al. reported high rates of polyhydramnios, induced labor and caesarean section, diminished fetal movement in a cohort of 86 French patients with PWS [11]. Gross et al. found decreased fetal movements, small for gestational age, asymmetrical intrauterine growth and polyhydramnios in an Israel cohort [19]. Gold et al. and Sign et al. reported significantly higher rates of caesarean section, polyhydramnios, decreased fetal movements, low birth weight, feeding difficulty, hypotonia and low Apgar scores in two cohorts of PWS patients in USA [13, 14].

Hence, differential diagnosis of PWS should be highlighted if infants having following perinatal factors including polyhydramnios, intrauterine decreased fetal movements, caesarean section, low birth weight, feeding difficulty, hypotonia and failure to thrive. Besides polyhydramnios, we also found a far higher rate of oligohydramnios (18.7%) in our cohort comparing with the general population (0.4–4.0%) in China. The accurate mechanism of polyhydramnios and oligohydramnios is still unclear although reduced fetal swallowing is regarded as a cause of polyhydramnios. High rate of oligohydramnios in this cohort may be due to the high rate of premature rupture of memberanes. Further study for the mechanism of amniotic fluid disorder is required. Moreover, caesarean section rate was higher in our cohort comparing with normal populations [7]. The high caesarean section rate in this cohort was due to the high rate of obstetric complications in PWS including abnormal amnion (61.2%), decreased fetal movements (87.9%), premature rupture of membranes (9.7%) or abnormal fetal heart rate/rhythm [20–22] although the data of fetal heart rate was not recorded in our study. High caesarean section rates may also be associated with the concepts and selection of pregnant women in China.

Considering neonatal complications, failure to thrive, low birth weight, feeding difficulty and hypotonia were most common in our study. Moreover, rate of asphyxia was higher in our cohort than that in general population in China (32.1% vs. 6.3%) [6]. This may be associated with higher ratio of intrauterine abnormal fetal heart rate/rhythm and decreased fetal movements, hypotonia and weak cry after birth, which may be considered as intrauterine or intrapartum asphyxia and higher ratio of preterm in our cohort as well. More than 99% of the infants had feeding difficulty requiring tube feeding and about 70% of the infants ever used feeding tube during hospitalization. Tube feeding was discontinued in 98% of these infants by their parents. Parents refused to use tube feeding at home, and they preferred using silicone bottle, spoon or even a syringe for feeding PWS infants which could not reach the effect of tube feeding. As a result, the rate of failure to thrive (94.5%) during the first months of life in this cohort of patients was higher than that reported by Sign et al. (77%) [14]. The possible reasons for refusing tube-feeding at home may be lacking education of tube-feeding from healthcare workers, in which parents are not familiar with home tube feeding techniques. Therefore, it is very important to strengthen feeding knowledge and ability of parents of PWS patients in China.

Differences between the deletion and UPD subgroups are still conflicting in literature. Gillessen-Kaesbach et al. and Whittington et al. showed that UPD patients had a significantly higher birth weight and maternal age than that of deletion patients [12, 22]. Vise versa, Gunay-Aygun et al. observed a significantly lower birth weights and lengths in the UPD group than those in the deletion group [22]. Dudley and Muscatelli found a higher rate of induced labor, premature labor and higher maternal age in the UPD group, and a high rate of low birth weight in the deletion group [11]. However, Sign et al. only found higher maternal age and pre-pregnancy weight in the UPD group of a large cohort containing 355 PWS patients from USA [14]. Whittington et al. found significant difference of maternal age and birth weight between different genetic subtypes [12]. In our study, higher maternal age and pre-pregnancy weight were noted in the UPD subgroup than that in the deletion subgroup. However, the rate of hospitalization at neonatal period and feeding tube use was higher in the deletion group with marginal differences (0.05 < *P* < 0.15). These results above implied that patients with deletion genetic type may have more difficulty in feeding and are prone to be hospitalized at neonatal period. Large cohort study is still needed to confirm these findings.

The mechanism of PWS still remains unknown. We observed a high maternal age of PWS patients, especially in the UPD group, which was also reported in several other reports [12, 14, 23]. These results imply that the advanced maternal age is associated with PWS resulting from non-disjunction at meiosis 1. Moreover, we also found that mothers of PWS patients had higher pre-pregnancy BMI than those in the general population and higher pre-pregnancy weight in the UPD group, which was also reported in a large cohort of 355 PWS patients from USA [14]. These suggested that high maternal age and overweight may be the risk factors of PWS. Further studies for the mechanism of PWS are required.

## Conclusions

In conclusion, our study highlighted the perinatal features in a large cohort of Chinese patients with PWS. We found high incidence of decreased fetal movements, polyhydramnios and delivery by caesarean section, and higher maternal age comparing with the general population. Neonatal features found in our cohort included low birth weight, birth asphyxia, failure to thrive, feeding difficulty, weak cry and hypotonia. We demonstrated that mothers of patients with UPD had higher maternal age, pre-pregnancy weight comparing with the deletion group. Most patients required tube feeding during hospitalization, however tube feeding was discontinued by their parents after discharge at home. Nutrition deficiency was a serious problem in infants with PWS. Home tube-feeding instruction should be carried out by parents of PWS patients in China. Our study is helpful to better understand the perinatal features of PWS in China, which will benefit for early diagnosis and treatment. Moreover, our results provide valid evidence for further research and promote PWS screening into the newborn screening program in China.

## Data Availability

Data can be accessed at https://dataverse.harvard.edu/privateurl.xhtml?token=c8c95002-a4b4-4aa8-b65d-8e47fac1ea77

## Abbreviations

ANCOVA: analysis of covariance
BMI: body mass index
PWS: Prader-Willi syndrome
UPD: uniparental disomy

## References

[1] Driscoll DJ, Miller JL, Schwartz S, Cassidy SB (1993). Prader-Willi syndrome. In: Adam MP, Ardinger HH, Pagon RA, Wallace SE, bean LJH, Stephens K, Amemiya a, editors. GeneReviews®[internet].

[2] Goldstone AP, Holland AJ, Hauffa BP, Hokken-Koelega AC. Tauber M, Speakers contributors at the second expert meeting of the Comprehensive Care of Patients with PWS. Recommendations for the diagnosis and management of Prader-Willi syndrome. J Clin Endocrinol Metab 2008;93(11):4183–4197. 10.1210/jc.2008-0649 18697869

[3] Cassidy SB, Schwartz S, Miller JL, Driscoll DJ (2012). Prader-Willi syndrome. Genet Med.

[4] Bachere N, Diene G, Delagnes V, Molinas C, Moulin P, Tauber M (2008). Early diagnosis and multidisciplinary care reduce the hospitalization time and duration of tube feeding and prevent early obesity in PWS infants. Horm Res.

[5] Bar C, Diene G, Molinas C, Bieth E, Casper C, Tauber M (2017). Early diagnosis and care is achieved but should be improved in infants with Prader-Willi syndrome. Orphanet J Rare Dis.

[6] Xie X, Kong BH, Duang T, editors. Obstetrics and gynecology, the 9th ed. Beijing People's Medical Publishing House, 2018.

[7] Boerma T, Ronsmans C, Melesse DY, Barros AJD, Barros FC, Juan L (2018). Global epidemiology of use of and disparities in caesarean sections. Lancet..

[8] Born Too Soon: The Global Action Report on Preterm Birth. https://www.who.int/pmnch/media/news/2012/preterm_birth_report/en/ Accessed 15 Dec, 2018.

[9] An Analysis Report of National Health Services Survey in China, 2013. http://www.nhfpc.gov.cn/ewebeditor/uploadfile/2016/10/20161026163512679.pdf Accessed 15 Dec, 2018.

[10] Wang WP (2018). Pediatrics, the.

[11] Dudley O, Muscatelli F (2007). Clinical evidence of intrauterine disturbance in Prader-Willi syndrome, a genetically imprinted neurodevelopmental disorder. Early Hum Dev.

[12] Whittington JE, Butler JV, Holland AJ (2008). Pre-, peri- and postnatal complications in Prader-Willi syndrome in a UK sample. Early Hum Dev.

[13] Gold JA, Mahmoud R, Cassidy SB, Kimonis V (2018). Comparison of perinatal factors in deletion versus uniparental disomy in Prader-Willi syndrome. Am J Med Genet A.

[14] Singh P, Mahmoud R, Gold JA, Miller JL, Roof E, Tamura R (2018). Multicentre study of maternal and neonatal outcomes in individuals with Prader-Willi syndrome. J Med Genet.

[15] Lionti T, Reid SM, White SM, Rowell MM (2015). A population-based profile of 160 Australians with Prader-Willi syndrome: trends in diagnosis, birth prevalence and birth characteristics. Am J Med Genet A.

[16] Angulo MA, Butler MG, Cataletto ME (2015). Prader-Willi syndrome: a review of clinical, genetic, and endocrine findings. J Endocrinol Investig.

[17] Butler MG (2017). Benefits and limitations of prenatal screening for Prader-Willi syndrome. Prenat Diag.

[18] Gross N, Rabinowitz R, Gross-Tsur V, Hirsch HJ, Eldar-Geva T (2015). Prader-Willi syndrome can be diagnosed prenatally. Am J Med Genet A.

[19] Fong BF, De Vries JI (2003). Obstetric aspects of the Prader–Willi syndrome. Ultrasound Obstet Gynecol.

[20] Geysenbergh B, De Catte L, Vogels A (2011). Can fetal ultrasound result in prenatal diagnosis of Prader-Willi syndrome?. Genet Couns.

[21] Gillessen-Kaesbach G, Robinson W, Lohmann D, Kaya-Westerloh S, Passarge E, Horsthemke B (1995). Genotype-phenotype correlation in a series of 167 deletion and nondeletion patients with Prader-Willi syndrome. Hum Genet.

[22] Gunay-Aygun M, Heeger S, Schwartz S, Cassidy SB (1997). Delayed diagnosis in patients with Prader-Willi syndrome due to maternal uniparental disomy 15. Am J Med Genet.

[23] Matsubara K, Murakami N, Nagai T, Ogata T (2011). Maternal age effect on the development of Prader-Willi syndrome resulting from upd(15)mat through meiosis 1 errors. J Hum Genet.

